# *Molluscum contagiosum* with atopic dermatitis: a clinical retrospective study of 2,278 children

**DOI:** 10.3389/fped.2025.1543309

**Published:** 2025-07-07

**Authors:** Tingying Li, Nan Gao, Zilu Zeng, Guili Fu

**Affiliations:** Wuhan Children's Hospital (Wuhan Maternal and Child Healthcare Hospital), Tongji Medical College, Huazhong University of Science & Technology, Wuhan, China

**Keywords:** children, *Molluscum contagiosum*, atopic dermatitis, treatment, curettage

## Abstract

**Objective:**

The dissemination of *Molluscum contagiosum* (MC), a prevalent pediatric cutaneous viral infection, is enhanced upon atopic dermatitis (AD) or compromised epidermal barrier function. However, the potential influence of AD on the course of MC remains controversial. This study aimed to evaluate the influence of AD on MC treatment outcomes.

**Methods:**

In this clinical retrospective study, we enrolled children with MC and divided them into control (patients with MC alone) and observation (patients with both MC and AD) groups. Parameters such as sex, age, treatment sessions, and treatment duration were recorded for all patients. Efficacy endpoints were defined as complete clearance, with no further MC treatment required for half a year. Chi-squared and *Z*-tests were performed to compare the clinical and demographic parameters between the groups.

**Results:**

Among 2,278 patients, 1,931 (84.77%) had MC alone, and 347 (15.23%) had concurrent MC and AD. Significant differences were observed in treatment sessions (*p* < 0.05) between the two groups. However, sex, age, and treatment duration did not differ significantly between the groups (*p* > 0.05).

**Conclusion:**

AD increased the number of treatment sessions of MC in children but was not closely related to sex, age, or treatment duration.

## 1 Introduction

*Molluscum contagiosum* (MC) is a prevalent viral infection in children, primarily affecting the differentiation of keratinocytes. This results in the formation of discrete, smooth, firm, waxy papules, often exhibiting a characteristic central dimple or umbilication (1). The incubation period for *Molluscum contagiosum* virus (MCV) typically spans from 2 to 6 weeks (2). Although MC is generally self-limiting, with lesions typically resolving within 1 year, active treatment is recommended to avoid discomfort, such as itching, and address concerns regarding transmission and autoinoculation (3, 4). A large variety of treatments for MC are available, including curettage, topical medications, and cryotherapy, but none have been approved by the Food and Drug Administration (FDA) (5). However, taking efficacy, cost, adverse effects, ease of use, and availability into consideration, curettage is the preferred option in pediatric patients.

MCV enters the epidermis via direct contact with infected skin. Scratching, often due to discomfort, facilitates viral autoinoculation, promoting further spread (6). Consequently, conditions such as atopic dermatitis (AD), ichthyosis, or other itchy dermatoses heighten susceptibility to MCV infection and enhance the likelihood of clinical manifestation (7). Notably, the prevalence of AD has risen steadily in recent years. However, the relationship between AD and MC remains controversial (8–10). Therefore, in the present study, we aimed to explore whether AD affects the treatment duration and efficacy of MC.

## 2 Materials and methods

### 2.1 Data collection

This observational, retrospective study was conducted at the pediatric dermatology outpatient clinic of the Department of Wuhan Children's Hospital. The data were collected between December 2021 and December 2023. Eligible participants included patients of both sexes, aged between 1 month and 18 years, with a clinical and dermoscopic diagnosis of MC. The clinical diagnosis of AD was established using Yao's diagnostic criteria (11). Patients with recent use of oral glucocorticoids, immunosuppressants, and dupilumab or those with organ transplants were excluded.

### 2.2 Treatment methods

Non-inflammatory lesions were treated with curettage, while lesions with pronounced inflammatory (characterized by visible erythema and pustule) received topical antibiotics for 1 week prior to curettage. Owing to the variable incubation period of MC, progressive decline in immune function, and frequent autoinoculation, MC lesions typically appear in clusters. This characteristic often necessitates multiple treatment sessions.

In this study, children with both MC and AD maintained their previous topical treatment regimens, including corticosteroids (applied to non-lesional areas) and moisturizers. The primary efficacy endpoint was defined as complete lesion clearance with no requirement for additional MC treatment over a 6-month follow-up period.

### 2.3 Study groups

Children were divided into two groups: control, which included patients with MC without AD, and observation, which included patients with both MC and AD.

### 2.4 Primary and secondary outcomes

The primary outcomes of the present study were the final treatment outcome of MC in the two groups, including the total treatment duration and number of sessions. The secondary outcome was to explore whether age and sex influence the incidence of AD combined with MC.

### 2.5 Statistical analysis

IBM SPSS Statistics, version 30.0 (IBM Corp, Armonk, NY, USA) was used for data analysis distribution. *χ*^2^ test was used to analyze the differences between the groups, while *Z*-test was used to compare differences between treatment sessions. *P* < 0.05 was considered statistically significant.

## 3 Results

During the study period, 2,295 children were diagnosed with MC. After excluding children receiving oral glucocorticoids or immunosuppressants (*n* = 3), those undergoing dupilumab therapy (*n* = 12), and prior organ transplant recipients (*n* = 2), the final cohort comprised 2,278 participants.

### 3.1 Characteristics of children in the control and observation groups

The characteristics of the patients are presented in the tables. The overall cohort (*n* = 2,278) had a mean age of 45 months (median, 39 months). The control group (*n* = 1,931) was older (mean age, 49 months; median, 45 months) compared with the observation group (*n* = 347; mean age, 41 months; median, 34 months) (Table 1).

**Table 1:** Mean and median age of the children in the study.

Groups	*N*	Mean (months)	Median (months)
Control	1,931	49	45
Observation	347	41	34
Control + observation	2,278	45	39

The control group comprised 1,168 males (60.5%) and 763 females (39.5%). Age distribution was as follows: 361 children (18.7%) aged ≤24 months, 1,055 (54.6%) aged 24–72 months, and 515 (26.7%) aged >72 months. Most children required only one treatment session (73.9%, *n* = 1,428), while fewer required multiple sessions (two sessions, 16.8%, *n* = 325; three, 5.3%, *n* = 102; four, 2.3%, *n* = 43; five, 1.0%, *n* = 20; more than five, 0.7%, *n* = 13). Among patients requiring multiple treatments (*n* = 503), cure rates by duration were as follows: ≤1 month (27.1%, *n* = 136), 1–3 months (23.5%, *n* = 118), 3–6 months (25.5%, *n* = 128), 6–12 months (18.5%, *n* = 93), and >12 months (5.4%, *n* = 27) (Table 2).

**Table 2:** Characteristics of children with control and observation groups.

Characteristics	Control (*N*/%)	Observation (*N*/%)	*χ*^2^ value	*p*-value
Sex				
Male	1,168 (60.49%)	196 (56.49%)	0.328	>0.05
Female	763 (39.51%)	151 (43.51%)		
Age (months)				
0–24	361 (18.70%)	62 (17.87%)	2.660	>0.05
24–72	1,055 (54.63%)	224 (64.55%)		
>72	515 (26.67%)	61 (17.58%)		
Treatment sessions				
One session	1,428 (73.95%)	164 (47.26%)	19.292	<0.05
Two sessions	325 (16.83%)	71 (20.46%)		
Three sessions	102 (5.28%)	55 (15.85%)		
Four sessions	43 (2.27%)	29 (8.36%)		
Five sessions	20 (1.04%)	18 (5.19%)		
More than 5 sessions	13 (0.72%)	10 (2.88%)		
Treatment duration (months)				
–1	136 (27.05%)	31 (16.94%)	6.116	>0.05
≥1–3	118 (23.51%)	53 (28.96%)		
≥3–6	128 (25.46%)	61 (33.33%)		
≥6–12	93 (18.50%)	22 (12.02%)		
≥12	27 (5.38%)	14 (7.65%)		

The observation group included 196 males (56.5%) and 151 females (43.5%), with age distribution of ≤24 months (17.9%, *n* = 62), 24–72 months (64.6%, *n* = 224), and >72 months (17.6%, *n* = 61). Treatment sessions required were as follows: one (47.3%, *n* = 164), two (20.5%, *n* = 71), three (15.9%, *n* = 55), four (8.4%, *n* = 29), five (5.2%, *n* = 18), and more than five (2.9%, *n* = 10). For multiple-treatment patients (*n* = 183), cure duration distribution was as follows: ≤1 month (16.9%, *n* = 31), 1–3 months (29.0%, *n* = 53), 3–6 months (33.3%, *n* = 61), 6–12 months (12.0%, *n* = 22), and >12 months (7.7%, *n* = 14) (Table 2).

### 3.2 Differences between the control and observation groups

The *χ*^2^ tests revealed no significant differences in sex or age distributions between groups (*p* > 0.05). However, the number of treatment sessions significantly differed (*p* < 0.05) (Table 2).

The *Z*-test was used to further analyze differences among treatment sessions. Statistical significance was observed between groups for all treatment sessions (*p* < 0.05), except for two sessions (Table 3).

**Table 3:** Statistical significance of treatment session with control and observation groups.

Treatment sessions	Control (*N*/%)	Observation (*N*/%)	*Z*-test	95% CI	*p*-value
One session	1,428 (73.94%)	164 (47.16%)	9.978	0.211–0.323	<0.05
Two sessions	325 (16.86%)	71 (20.74%)	−1.643	−0.082 to 0.009	>0.05
Three sessions	102 (5.27%)	55 (15.63%)	−7.155	−0.145 to 0.066	<0.05
Four sessions	43 (2.22%)	29 (15.63%)	−6.010	−0.091 to 0.031	<0.05
Five sessions	20 (1.03%)	18 (5.11%)	−5.559	−0.065 to −0.018	<0.05
More than five sessions	13 (0.67%)	10 (2.84%)	−3.789	−0.040 to −0.004	<0.05

## 4 Discussion

In the present study, we investigated the effects of AD on the number of sessions and duration of treatment in patients with MC. Our cohort study demonstrated that while AD increased the MC treatment sessions in children, it was not closely related to sex, age, or treatment duration.

Various risk factors for MC, including multi-child families, swimming, AD, and filaggrin (*FLG*) gene mutations, have been identified (12). The immunological interplay between AD and MC warrants particular attention: MCV encodes a protein homologous to interleukin-18 (IL-18)-binding protein (13), while AD-driven Th1-cell responses produce interferon-gamma (IFN-γ) and IL-18, creating a proinflammatory microenvironment that may theoretically facilitate MC persistence (10, 14, 15). Conversely, other studies have demonstrated no significant differences in the incidence or recurrence rate of MC in patients with both MC and AD (16). However, the number of papules and the severity of itching symptoms are often aggravated in children with AD (17). Since we did not investigate MC incidence, further studies are needed to determine whether AD increases MC incidence and its underlying mechanism.

Our study identified concurrent MC and AD in 15.2% of all patients, aligning with previously reported rates of 15%–24% (16, 18, 19) but significantly below the estimate of 40% reported in another study (4). Sex distribution showed no significant differences between control and observation groups, which is consistent with prior research (20). The cohort age range spanned 4–148 months (mean, 48 months; median, 41 months), matching established epidemiological data (14). MC incidence peaked between 24 and 72 months of age, contrasting with reports of highest incidence at 6 years of age (12, 21). Notably, the comparable mean (48 vs. 47 months) and median ages (41 vs. 40 months) between the two groups suggest minimal age-dependent effects of AD on MC prevalence.

Currently, the need for active treatment in patients with MC is controversial, given the self-limited course of infection, the large number of therapeutic alternatives available, and the lack of evidence to deﬁne the best therapy (6). Although MC is self-limiting, active treatment is often recommended for discomfort, further autoinoculation, or transmission concerns (4). Parents worry about prolonged restrictions on group activities. Current MC therapies fall into two broad categories: mechanical (e.g., cryotherapy, curettage, and CO₂ laser) and medical (e.g., chemical agents like cantharidin and trichloroacetic acid; immunomodulators such as imiquimod 5% and interferon-α; and antivirals such as cidofovir). However, medical therapies in pediatric patients often exhibit slow efficacy and pose challenges in dosage control. Mechanical approaches also present limitations: Cryotherapy may induce blistering, while CO₂ laser therapy is cost-prohibitive for many. Curettage, although can lead to bleeding and scarring, is not only cost-effective and rapid but also provides direct visual confirmation of complete *Molluscum contagiosum* content removal, guaranteeing therapeutic effectiveness. In China, patient preferences strongly favor affordable, rapid-resolution treatments. At our institution, curettage is the predominant intervention for pediatric MC patients due to its balance of efficacy, accessibility, and cost-effectiveness.

The treatment duration for MC typically lasts up to 1 year, and recent studies have demonstrated that AD does not significantly affect the outcome of MC treatment (8, 22, 23). Our analysis of the number of treatment sessions and duration revealed that while most children with MC alone were cured after a single session, over 80% of those with MC and AD achieved resolution after three treatment sessions. Furthermore, the recurrence rate of MC was higher in children with AD than in those without AD, which is inconsistent with the findings of previous studies (9, 24). Three potential explanations emerge for this discrepancy: (1) variability in study endpoint definitions (21); (2) differences in cohort sizes and composition; and (3) concurrent AD treatments potentially influencing outcomes. Prior studies have suggested that long-term use of topical glucocorticoids and dupilumab can prolong the resolution time of MC, while short periods of topical steroids in severe AD cases may help alleviate itching (25–27). In this study, we excluded children who received dupilumab. Nevertheless, some patients with MC and AD continued using topical glucocorticoids to manage their AD. Unfortunately, most of the existing literature on this topic are retrospective articles, and the severity of AD and the use of topical glucocorticoids have not been systematically classified and explored, including in the present study, which may influence the results of the research. In addition, in our study, the distribution of treatment durations did not differ between the groups. This result is consistent with the mechanism by which MC and AD promote inflammation of the skin, as well as previous findings. Overall, our results indicate that the presence of AD affects MCV replication, increasing the recurrence frequency and the number of visits. However, considering that MCV has a self-limitation period of approximately 1 year, the MC infection process can be inhibited after multiple curettages of the infection sources and induction of inflammation, resulting in disease remission.

This study was strengthened by a large sample size, providing a comprehensive view of the effect of AD on final treatment outcomes in children with MC. But it has some limitations. First, the number of children with MC and AD was limited (196 boys and 151 girls), differing significantly from the population of children with MC alone. Second, several factors, including skin dryness and initial distribution or count of lesions, remain controversial and were not considered in this study, which potentially introduced bias. Thirdly, as a retrospective study, the stratification of AD severity in patients, as well as the regulation and monitoring of topical medications, remain incomplete. Finally, variations in the degree of parents’ understanding of the disease contributed to differences in the number of treatment sessions among the children. Therefore, future research on MC should consider the clinical manifestations in children at their first visit and emphasize health education for parents.

In conclusion, this study highlights the impact of AD on the outcomes of treatment for MC in children. However, given that assessing the morbidity of MC was beyond the scope of the study, AD may increase the recurrence rate of MC, without significantly affecting the entire disease course.

## Data Availability

The original contributions presented in the study are included in the article/Supplementary Material; further inquiries can be directed to the corresponding author.
